# Control cell death by endosomal trafficking of proton transistor nanophotosensitizers

**DOI:** 10.1093/lifemedi/lnac023

**Published:** 2022-07-21

**Authors:** Binlong Chen, Qiang Zhang, Jinming Gao, Yiguang Wang

**Affiliations:** State Key Laboratory of Natural and Biomimetic Drugs, School of Pharmaceutical Sciences, Peking University, Beijing 100191, China; Beijing Key Laboratory of Molecular Pharmaceutics and New Drug Delivery Systems, School of Pharmaceutical Sciences, Peking University, Beijing 100191, China; State Key Laboratory of Natural and Biomimetic Drugs, School of Pharmaceutical Sciences, Peking University, Beijing 100191, China; Beijing Key Laboratory of Molecular Pharmaceutics and New Drug Delivery Systems, School of Pharmaceutical Sciences, Peking University, Beijing 100191, China; Department of Pharmacology, Harold C. Simmons Comprehensive Cancer Center, University of Texas Southwestern Medical Center, Dallas, TX 75390, USA; State Key Laboratory of Natural and Biomimetic Drugs, School of Pharmaceutical Sciences, Peking University, Beijing 100191, China; Beijing Key Laboratory of Molecular Pharmaceutics and New Drug Delivery Systems, School of Pharmaceutical Sciences, Peking University, Beijing 100191, China

Regulated cell death plays an essential role in embryonic development, tissue homeostasis, inflammation, immunity, and various pathophysiological events. Drug-induced cell death is also important in the treatment of diseases. Increasing efforts have been dedicated to the development of medicines that can selectively target distinct cell death pathways for efficacious therapy in the diseased tissues with low toxicity in healthy tissues.

In cancer therapy, emerging evidence has shown that programmed cell necrosis, including necroptosis, ferroptosis, and pyroptosis, exhibits higher therapeutic potential including favorable immune response, than apoptosis, because of the intrinsic or acquired apoptotic resistance [1]. As a unique form of necrosis mediated by specific caspases, pyroptosis has been recognized as a critical effector mechanism in innate and adaptive immune responses in recent years. It is still challenging to develop controllable and tumor-specific pyroptosis inducer for safe and effective cancer therapy. Since the identification of gasdermin family as the executor of pyroptosis by Shao Lab [2], several nanoparticle-based strategies have been developed for the induction of pyroptosis in cancer cells. Recently, reported nanoparticles mainly trigger pyroptosis by eliciting reactive oxygen species (ROS) stress in lysosomes (Ly) [3], and little attention has been paid to investigating the ROS stress and the consequent pyroptotic efficacy in the early endocytic organelles.

Endocytic organelles play a critical role in many cellular physiological processes such as protein/lipid metabolism, nutrient sensing, and cell survival. Along the endocytic pathway, progressive acidification separates uncoupling of receptor–ligand pairs (e.g. at pH 6.5) and activation of proteases for protein/lipid degradation (e.g. pH < 5.0) into endosomes and Ly, respectively. Luminal pH is a hallmark of endosome/Ly maturation that impact cellular and physiological functions at different stages of maturation.

**Figure 1.**
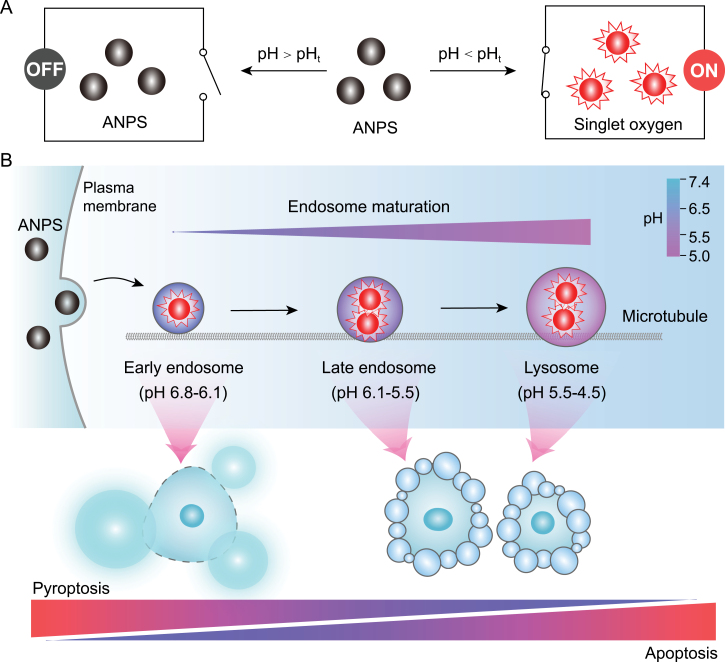
The tunable pyroptosis and apoptosis evoked by ANPS through endosome maturation. (A) Schematic illustration of proton transistor nanophotosensitizers enabling discrete amplification of acidotic signals into a binary output of singlet oxygen. (B) The ANPS with different pH transition can spatiotemporally target and induce ROS stress in distinct endocytic organelles (0.2 pH interval) during endosome maturation. ROS stress in different endocytic organelles can evoke distinct cell death patterns. The EE-targeted ROS stress prefers to induce robust pyroptosis, while ROS stress in LEs and Ly trends to evoke apoptosis.

Recently, a small acid-activatable nanophotosensitizer (ANPS) library that can spatiotemporally deliver ROS into distinct endocytic organelles was reported to selectively evoke pyroptotic cancer cell death while sparing normal tissues [4]. The library was built on the proton transistor concept manifested by ultra-pH-sensitive (UPS) nanoparticles [5], which demonstrate sharp micelle-to-unimer phase transitions across a narrow pH range, enabling discrete amplification of acidotic signals into a binary threshold output (Fig. 1A). Previous work has shown the digitization of endosomal/lysosomal pH at a single organelle resolution [6], and image and perturbation strategy to investigate lysosomal and autophagy biology [7]. These ANPS nanoprobes were further functionalized with a series of photosensitizer-conjugated UPS copolymers that exhibited 300-fold singlet oxygen generation amplification at distinct pH transitions (pH_t_) from 6.9 to 5.3. Therefore, the ANPS with different pH_t_ can temporally activate in early endosomes (EEs), late endosomes (LEs), and Ly, respectively, succeeded to divide the entire endosome maturation pathway into 10 regions (pH ranging from 7.0 to 5.0), and precisely induced ROS stress in specific endocytic organelles. Enabled by the ANPS nanotechnology, this study reveals that the endosome maturation process can regulate the cell death pattern and pyroptosis-inducing activity of nanomedicine-derived ROS stress (Fig. 1B). EEs displayed dramatically higher sensitivity to ROS stress than other endocytic organelles. The pyroptotic cell killing ability of ROS in EE was up to 40-fold higher than that of equal dose of ROS in LE and Ly with broad applicability in a series of cancer cells. Moreover, the ROS stress generated in LE and Ly tended to induce apoptosis instead of pyroptosis.

The subcellular components, including different endocytic organelles, exhibits heterogeneous sensitivity to ROS stress due to the location-dependent signaling. Many membrane receptors are transported to the EE membranes through cellular internalization, whereas these receptors are quickly attenuated on membrane of LEs and Ly due to the receptor recycling and lysosomal degradation [8]. Hence, the EE is an essential target for ROS stress and signal transduction. However, it is extremely hard to probe the signaling events in EEs because of the preceded signaling events on the plasma membrane and very short duration in this organelle (10–30 min). Although, optogenetics and conformational activation-based methodologies have been exploited to distinguish the signal pathways in EEs from those on plasma membrane [9, 10], there has been no study reported to differentiate the signal transduction among the whole endosome maturation pathway.

A vast range of studies have revealed that the photo-induced ROS can trigger several cellular stresses and lead to cell apoptosis or necrosis. However, the underlying mechanism of the cell death pathway remains unknown. Because of the Always-On design, previous nanomaterials can induce persistent signaling transduction on plasma membrane, which conceals the following signaling events on the endosome maturation pathway. Due to the tunable pH_t_ (6.9–5.3), ultra-pH responsiveness (ΔpH_ON/OFF_ < 0.25 pH), ultra-fast pH-induced activation (5 ms), and super-high ROS amplification (~300-fold), the ANPS platform provides a suitable toolkit to clarify the signal transduction elicited by ROS stress in each tiny region of the entire endosome maturation pathway. Combined with the pulse-chase method, this in-depth investigation demonstrated that the phospholipase C (PLC) in plasma membrane could transport into EE along with the internalization of ANPS, and thus specially activated by the ROS-induced lipid peroxidation on the membrane of EE. The activated PLC signaling further initiated the mitochondria-based caspase-3 pathway, and cleaved gasdermin E (GSDME) to execute robust pyroptosis. Whereas, the GSDME-mediated pyroptosis was diminished in LE and Ly due to the quick sorting of PLC during endosome maturation. Instead, the LE/Ly-located ROS stress can promote lysosome membrane permeabilization to release lysosomal cathepsins and trigger cell apoptosis. Furthermore, the pyroptosis-inducing activity and tunability of this nanotuner displayed a positive correlation with the GSDME expression of tumor cells, and upregulation of GSDME expression by demethylation further facilitated the ANPS-mediated pyroptosis. Altogether, the ANPS-based pyroptosis nanotuner efficiently controls the cell death via endosome maturation.

This pyroptosis-tunable nanotechnology exhibits great potential for safe and effective cancer therapy. On one hand, the high internalization capacity of cancer cells and pH-activatable design of ANPS can induce much higher level of ROS stress in tumor tissues than the surrounding healthy tissues. On the other hand, the final accumulation of the nanoprobe into Ly can efficiently reduce the toxicity to normal tissues due to the poor induction of pyroptotic cell death by ROS stress in LE/Ly. In consideration of the nonbiodegradability of the ANPS, more effort needs to be paid to develop safe and biodegradable pyroptosis nanotuners for clinical application. This study uncovers that EE is the most appropriate target for organelle stress-based cell death, and fills the gap that endosome maturation pathway plays an important role in cell death signaling. It not only provides guidelines for ROS stress-based cancer therapy, especially for photodynamic therapy, but also offers new insights into how to engineer nanocarriers to regulate specific signaling transduction in endocytic organelle, such as the toll-like receptor and neurokinin 1 receptor.
